# Supplementary material for: RNAi-Mediated Knockdown of Transcription Factor E93 in Nymphs of the Desert Locust (Schistocerca gregaria) Inhibits Adult Morphogenesis and Results in Supernumerary Juvenile Stages
Source: Int J Mol Sci. 2020 Oct 12;21(20):7518. doi: 10.3390/ijms21207518 (PMC7590052; doi:10.3390/ijms21207518)
Supplement: Supplementary file 1 [file ijms-21-07518-s001.pdf]

RNAi-mediated knockdown of transcription factor E93 in nymphs of the desert locust (*Schistocerca gregaria*) inhibits adult morphogenesis and results in supernumerary juvenile stages

Marijke Gijbels, Elisabeth Marchal[§], Thomas Wolf Verdonckt, Evert Bruyninckx, Jozef Vanden Broeck[*]

Research group of Molecular Developmental Physiology and Signal Transduction, KU Leuven, Zoological Institute, Naamsestraat 59 box 2465, 3000 Leuven, Belgium

[§]Current address: Life Science Technologies, Imec, Kapeldreef 75, B- 3001 Leuven, Belgium

[*]**Correspondence:**

Jozef Vanden Broeck: jozef.vandenbroeck@kuleuven.be

Marijke.Gijbels@kuleuven.be
Elisabeth.Marchal@imec.be
ThomasWolf.Verdonckt@kuleuven.be
Evert.Bruyninckx@kuleuven.be
Jozef.VandenBroeck@kuleuven.be

ATGGCGGAGTGCTCCTATGCTCGCTGTGTTCAGGAGCGCCGAGCAATCAAAAAAGAGCTGCAAAGATGGACG
AAGAATATGGTTTTCGTAGTAGGTTTGGAACGGGTAGCGGAGGAGTTAATGGGCAGAAGGAAATGGAAACA
TTATCAGGAGTCAGTTTTGAGAAGTAGACCGCAAGCAGACATAGAACCCAGCAGCAGTGATTGGCAAGTTGA
AGACAAATGCTGCTTTTGCGACGGTGGGATGTTCCTGAGACCAGCAACAAGTCCACAGTCAGACAGCTGCAGT
AGTTCCAACAGCCACAGCAGTTTTCACGAAACTCAGTCGACAGTAGCAGCAGACAGTTCCCCTCCCAGGCTGG
CGATGACAACACTCGAACCGGTCACGTCGCTGGCCGCGTCACTGGCAGCTGTCCCGACCTACAGCCCGGGACC
CGCACAGGGGCACTCCGCAATGGCAACCACCCCTCCAGCAGGCCTCTCTCTCTACCCCACAGTAGCACGCCATC
GGACACCTGCGGGACTCTTCCCTCCGTGGTACCTGTCACCGACGGCACCTGCTCCGAATGTTCGATCGGAAGG
AAAGACTGAACCGGAAACTCCACCCGTCGTCGTATCCCTCCCTGCATCTACACCGCTGTCGGTGCCGTCAGCTG
CGGCGAGTGAGCAGCCTTTGGACTTGAGTGCCAAAGCCAAGGAAGAAGCATCTGCACCCAGCACCTCACCCGT
GCCTCAGCCTTTGGGATTGGCAACTTTGAGTTTGGACAGCAAGCAGATATTCAAGGCGAAACCAAGAATGAGT
GCAGTGGCAGGTCGAAGGACATACACAGAAGAAGAGCTTCAAGCTGCTCTGAGAGATATCCAGAGTGGAAAA
TTAGGAACTCGGCGTGCAGCTGTTATTTATGGTATACCACGCTCCACACTTCGGAACAAAGTGTACAAGTTGGC
TATGGAGCGTGAACGTGACGCACACTTAGTGGTTCCTACAGTAGAGGAAAGAGACCTGTCTGGCGCTGAGGA
TGAGAAGGAAGTTGAACGTGCTTTAAGTCGTCCTCTTCTTTCTGTTGACGATCTTCTGCGATTATCTGTGCTAGA
TGGGGATGCTCTTAGAGTTCTTCTGGAGGAAGTTTCTGCAGGACACAATCGTGAAGCAAGTGAGTCAGCATCT
TCCCCAGTGTTTCCACAATCTAGTGAACTTTGGCATGGTCTAGAACAGAGTGCATTGGGACCTTACATCTCCCA
GCTTCTGATAGCTGGAAGTGCTCAAGATCCTTGCCATGCTGAAAGCGGAGGTACACTCCCAAAATTTACTTCCC
CTCTTCTCCCCGAACTTGTCCGTCGCATGATGGCAGAGGAGCAGCAACAGCAGATAAAGAAGCTAGGTAGTGC
CAATAATTTGGAAGCAAATACAAATATAACATTGAATGGTGCTTCAGGTGATGAGATACAGAAGTTAGGTGAT
AGCGACAACAGTGCTGGGCCACCGAGAGTTCCATCTGCATCATCTCAAACAGACCCTGTGAAAGCTACAACAA
AAGTTGGGGAAGCAGATGAATCATCTGACACAGATGCAACAGCTTCCACTTCTAGAGTTGCCACACCACCAAA
TGTGATACTGAGAATTCCGTCCTTTAAACCAACTACGAAAAATGGTGTGGCTACTCCAGGAAGTGGAAGTTTG
CCTTCTAGTGAAATTCCATTCCAGACTGTTGGAGTTCCTGCATTTCCTGGCCAGGAATCTAGTCATGTATTGAAC
AGCAGCAGTAACAATAGTAATGAGTCTTGTTCACCACCAGCATCTAACCTTGTAGGAAAAGGGATTGGGGTCA
GTTTACGTGATGTTATAGCCAAAAGCATTAGTCAGAAGTTTCAACCAACTGATCTTCCAATGTCACAGAAGCTC

TTATTATCAGCAGATGAACAACCCCCATTTAAGAGAGGTCGTTTTACTCCTCCATTAAATACAAATATCACATCG
TGCACTATAAAACACAATAACAATAACAACACTAATGTTGATGATAAAAACAAGACATCCTCTGCAGGTGGAA
AAACTTCATCAAATTCTACTGGCAAAGGAACCCGGCCAAAACGTGGAAAATACCGTAATTATGATCGTGATAG
TTTGGTAGAAGCTGTTAGAGCAGTGCAGAGGGGTGAAATGAGTGTCCATCGGGCAGGATCATATTATGGTGT
TCCACATTCAACATTAGAATACAAGGTGAAAGAGAGACATTTGATGAGACCTCGGAAGAGGGAGCCCAAGCA
ACAACAGTCTGATGAAGTTAAAAGGAAAGATGAATCTGGGACTTCAATTCCAAGGAGTACAGCAACAACCTCA
TCAGCACTGGCAAATGATAAACTTAAAACTTTGCCTAAACCTCCCAAGACTGCCTTTAATTCACCTGCTCCATTA
CCTGGTGCACCAAATGGTCTGAAAATACCACCCTTGTTTGATCCCAGTTTATCTCTTGCCGCATATGCTAATGTT
ACAACAGCCTTTCCATTTTGGCCTGGTCCATTTCATGCCTTACCAGTTCCAGATTTTACCACTCCTACTGGTAGCT
TTCCTCCAAATCCTGAACATTTCTTTGCACCACAAATGATACACAGGCTACAAGATGAAACTAGGACTGCACCA
CGAACGAGCCCCTCTTCAGTAGTTCCTCCTCTTGGGAAGACTGCACGAGAGATGGCAGCATCTTTGTATGATG
GAACAGGAGCTAATGGTAATTTTCTTGATGGAATAATTCGTTCAAGTCTTGAGATGGGTTTGCCATCATCCTCA
CCCAAAGAAGGCAGTACAGGTGTGGGAAATAGCGGAAAAATGTCTAACAAAGCACTCATTGACCAGCTTTGTC
GTAACAGTTGTGTCACTCCTCTGCCCAAAGCTCCGACACCTTTTAGTATGAAAGATTGCAGTACTAGTGTAAAT
AGCTCAGATGAAGAACCTGTAAAGCAAGAAAGTGTCATCCCGGAACAGCCCTTAGAATCTGCTTCTCCTGCAG
AGCCTGTAGTTTGTCTATCAAATTCATCCAGTGGCCTGCCAGAACATGACTGTAGAATAGATAGTGAACATGTT
ACTTCAGTTCCAGATGAAAAACATAGCAAGGACAGTGTTTTGCCACTGTCAGGGGTGCACTGTGTGTCATCAA
CTGAAGATAGTTCTGAATTATTTACTAAAGATTCAAGTGAATTCCATAAAGAACATGTTCATAATCCAACTGAA
GAAAGTGCCTGTGATAGTAAAACTGTAAAAATACTTGGTGATATTGAAGCTGCCAAAGACTTACTCCCTAAAG
AAGAAGGCAACGAGGATTCAGAACAAACTGAGGATACCAAATCAATGACGGTCAACTCATAG

Supplementary Figure S1: *SgE93* nucleotide sequence with primer sets indicated. Complete coding sequence of *S. gregaria E93* mRNA as found on NCBI (accession number LC215000.1) (Sugahara et al., 2017) with localisation of the different primers that were selected for production of the dsRNA construct or for performing qRT-PCR assays. Sequence regions highlighted in yellow indicate the position of the primer set that was used for generating the *dsSgE93* construct. Sequence regions highlighted in purple indicate the primer set used for qRT-PCR.

MAECSYARCVQERRAIKKELQRWTKNMVFVVGLERVAEELMGRRKWKHYQESVLRSRPQADIEPSSSDWQVEDK
CCFCDGGMFLRPATSPQSDSCSSSNSHSSFHETQSTVAADSSPPRLAMTTLEPVTSLAASLAAVPTYSPGPAQGHSA
MATTPPAGLSLYPTVARHRTPAGLFPPWYLSPTAPAPNVRSEGKTEPETPPVVVSLPASTPLSVPSAAASEQPLDLSA
KAKEEASAPSTSPVPQPLGLATLSLDSKQIFKAKPRMSAVAGRRTY**TEEELQAALRDIQSGKLGTRRAAVIYGIPRST**
LRNKVYKLAMERERDAHLVVPTVEERDLSGAEDEKEVERALSRPLLSVDDLLRLSVLDGDALRVLLEEVSAGHNREA
SESASSPVFPQSSELWHGLEQSALGPYISQLLIAGSAQDPCHAESGGTLPKFTSPLLPELVRRMMAEEQQQQIKKLG
SANNLEANTNITLNGASGDEIQKLGDSDNSAGPPRVPSASSQTDPVKATTKVGEADESSDTDATASTSRVATPPNVI
LRIPSFKPTTKNGVATPGSGSLPSSEIPFQTVGVPAFPGQESSHVLNSSSNNSNESCSPPASNLVGKGIGVSLRDVIAK
SISQKFQPTDLPMSQKLLLSADEQPPFKRGRFTPPLNTNITSCTIKHNNNNNTNVDDKNKTSSAGGKTSSNSTG**KGT**
RPKRGKYRNYDRDSLVEAVRAVQRGEMSVHRAGSYYGVPHSTLEYKVKERHLMRPRKREPKQQQSDEVKRKDE
SGTSIPRSTATTSSALANDKLKTLPKPPKTAFNSPAPLPGAPNGLKIPPLFDPSLSLAAYANVTTAFPFWPGPFHALPV
PDFTTPTGSFPPNPEHFFAPQMIHRLQDETRTAPRTSPSSVVPPLGKTAREMAASLYDGTGANGNFLDGIIRSSLEM
GLPSSSPKEGSTGVGNSGKMSNKALIDQLCRNSCVTPLPKAPTPFSMKDCSTSVNSSDEEPVKQESVIPEQPLESASP
AEPVVCLSNSSSGLPEHDCRIDSEHVTSVPDEKHSKDSVLPLSGVHCVSSTEDSSELFTKDSSEFHKEHVHNPTEESAC
DSKTVKILGDIEAAKDLLPKEEGNEDSEQTEDTKSMTVNS

Supplementary Figure S2: *Sg*E93 amino acid sequence with protein domains indicated. The amino acid sequence of *S. gregaria* E93 as found on NCBI (accession number BBA65756.1) with localisation of the different protein domains. The amino acid sequence regions highlighted in yellow and green indicate the CtBP-interaction motif and NR-box, respectively. The amino acid sequences indicated in bold represent the two HTH-DNA binding motifs, RHF1 (bold and underlined) and RHF2 (bold).

```
SgE93   MAECSYARCVQERRAIKKELQRWTKNMVFVVGLERVAEELMGRRKWKHYQESVLRSR-----------------------
BgE93   ----------------------------------------MGRRRWKQYQDSVLRSP-----------------------
DmE93   -----------------------MHISSYEISLERVAEECMGRRQWKHYQDKLTCSHLNIEEQQPIAIAGSEDEPSQYNH
Bm2E93  -------------------MYWYPRDRKRNRGLERVAEELMGRRKWKLYQDALIPKR-NEQDDSDDSMPSTDPPPALKIK
BmE93   ----------------------------------------MGRRKWKLYQDALIPKR-NEQDDSDDSMPSTDPPPALKIK
LdE93   ----------------------------------------MGRRKWKLYQESMSRSYLQPLNNNIKRSNPPEEDLDEYHH

SgE93   -----------------------------------------------------PQADIEPSSSDWQVEDKCCFC-DGGMF
BgE93   -----------------------------------------------------LRVDVEPRIQDWEPQEKCCMC-DGKPF
DmE93   SSKEISQSNPNHCKTENHRLEQQHNGSQLLEEEDSENNQTSHDSSRTPTPGATSTPSPPPEPIDWRPSAKCNFCVNGRLL
Bm2E93  TIEE----------INAPEDERPRVESDGNGQESKTSRPETILESLIKRPATQPKVEVLEEPADWKPPDKCYFCVDGEP-
BmE93   TIEE----------INAPEDERPRVESDGNGQESKTSRPETILESLIKRPATQPKVEVLEEPADWKPPDKCYFCVDGEP-
LdE93   HHKDYKQN------LHPHQADSEGNEPKTGGKEEEARGEGGGGDRAIEGHQKENEKDVD-RLKDWTPQDKCYFCVDGKLD

SgE93   LRPAT----SPQSDSCSSSNSHSSF-----------------HETQSTVAADSSPPRLAMTTLEP--------VTSLAAS
BgE93   FDSAT----SPAASDSGSTGTSSSL-----------------RETPPHEAAP------AMTTLQS--------VTSMAAS
DmE93   TVNAQGKLVAESAATATSSSTSNSHI----------------HQHDSDSNSSASLPHHISSSSSSNNNSSGNRARHIAAA
Bm2E93  --RATAEAAQPVGATSPASESDSSSV----------------SGTNSPAAAPPLLQHLLQLQAQN--------PQTIAQF
BmE93   --RATAEAAQPVGATSPASESDSSSV----------------SGTNSPAAAPPLLQHLLQLQAQN--------PQTIAQF
LdE93   SEHTTHGVLSPRPPDSDTSDSHSDSEVPASLTPTSRGLLNNNHHHHRHNVQHPVTPSSAMTTIEN--------V-----S

SgE93   LAAVPTYSPGPAQGHSAMATTP--PAGLSLYPTVARHRTPAGLFPP-WYLSPTAP----APNVRSEGKTEPETPPVVVSL
BgE93   IAAVAALSSSSGTGTTTNPLLPSQPSAMPFYP-------PPPHFPP-WYLSPPHSHLPGRPVLADAKQDPSATPPAVVHL
DmE93   SARATPAAATPANSLELYKLLTQRAAKMTSMDSMAAQLAQFSLLAD-FNLINSLASQQQQQQQQQIASAVTPTTSEVSAA
Bm2E93  QQMIAALT-ALGTGLV-----------------------PPPLTQA-WMMQRFAQQRHQADRLSESDKAAAPSSP-----
BmE93   QQMIAALT-ALGTGLV-----------------------PPPLTQA-WMMQRFAQQRHQADRLSESDKAAAPSSP-----
LdE93   MAALAAAALSGGASPVGTPAAP----HLPFYN-------PASVLTHNWYLANVVRFQNAAANDAAVDKAS----------

SgE93   PASTPLSVPSAAASEQPLDLSAKAKEEAS-APSTSPVPQPLGLATLSLDSKQI----FKAKPRMSAVAGRRTYTEEELQA
BgE93   PASS-------SASEQPLDLSAKTAA----VEIPSPAPPDPSLKVPSIDNKHI----FKAKPRMSTVAGRRTYTEEELQA
DmE93   AISPALKDTPSPSVDAPLDLSSK-------PSPNSSISGDVKSVRACAT---------------PTPSGRRAYSEEDLSR
Bm2E93  -----------SPVEQPLDLSAKSTSSTSGTPPPDPKFLDSRLRRTALDGA-------------SNSTGRRTYTEDELQS
BmE93   -----------SPVEQPLDLSAKSTSSTSGTPPPDPKFLDSRLRRTALDGA-------------SNSTGRRTYTEDELQS
LdE93   -----------GAGEQPLDLSKGGSNNNP-PATDRKVPLAINVRLPTLDTKHIFNSDFRAKPRMSAVAGRRTYTEDELQA

SgE93   ALRDIQSGKLGTRRAAVIYGIPRSTLRNKVYKLAMERERDAHLVVPTV----------EERDLSGAEDEKEVERALSRPL
BgE93   ALRDIQSGKLGTRRAAVIYGIPRSTLRNKVYKLALEKERDSHLVAPAVPKVEEE----DEKELSGAEEEREVEKALRKPL
DmE93   ALQDVVANKLDARKSASQHHEQRSILDNRLFKMKHHDQEQDHDG--------------DELEDSNDDAEAEVDSNASTPV
Bm2E93  ALRDIQSGRLGTRRAAVLYGIPRSTLRNKVNKF-----------------------------------------------
BmE93   ALRDIQSGRLGTRRAAVLYGIPRSTLRNKVNKF-----------------------------------------------
LdE93   ALRDIQSGKLGTRRAAVIYGIPRSTLRNKVYKLALERERESHINSSAPLKLEEEEAMDDDKELSGAEEEKEVEKALQAPL

SgE93   LSVD----DLLRLSVLDGDALRVLLEEVSAGHNREASESASSPVFPQSSELWHGLEQSALGPYI-SQLLIA--GSAQDPC
BgE93   LSME----DLVRFSVFKDAN------------------------YPDA---WGGLEHSALGPYV-AELLAAQKNPASTPG
DmE93   YPAEFARAQLRKLSHLSEHNGSDLGEDVD----RGSPKMGRHPACGNASANQGAPPSIPLDANVLLHTLMLAAGIGAMPK
Bm2E93  -------------------------------------------------------------------------GLVADNH
BmE93   -------------------------------------------------------------------------GLVADNH
LdE93   LSMA----DLYRFSG------------------REQPPDALKTLLQRG---KEGMNPSEMGPYI-KNLIMASQNILANQK

SgE93   H--AESGGTLPKFTSPLLPELVRRMMAEEQQQQIKKLGSANNLEANTNITLNGASGDEIQKLGDSDNSAGPPRVPSASSQ
BgE93   DKSPTSGDFLPKFPTPLLPEFVRRMMAEDKLQSFHP---------------NGAHADERE----------PER-------
DmE93   ---LDETQTVGDFIKGLLVANSGGIMNEGLLNLLSA-------------SQENSNGNASLLLQQQQHQQHHQQHHQQQQQ
Bm2E93  D--SD-----------------------------------------------------------------PDS-------
BmE93   D--SD-----------------------------------------------------------------PDS-------
LdE93   S--PEG--------TPMIPELMKMIAEEQLLKQQHQ---------------QYNNGDNERLLRPS-----PSN-------

SgE93   TDPVKATTKVGEADESSDTDATASTSRVATPP-NVILRIPSFKPT-TKNGV-----ATPGSGSLPSSEIPFQTVGVPAFP
BgE93   --------------PLRDQESPDPSPGMATPPSNVILKIPSFKPT-SKNGV-----ASSSGSTEPPPPLP------PPPP
DmE93   QQHVAAYRHRLPKSETPETNSSLDPNDASEDP---ILKIPSFKVSGPASSS-----SLSPGGLVGGHHHPLNNNNSLSIS
Bm2E93  -----------------DQDRAES------PP-SVILKIPTFPPPDDKSPS-----PATPVTTPITPLTPLISQPPPSLN
```

```
BmE93   -----------------DQDRAES------PP-SVILKIPTFPPPDDKSPS-----PATPVTTPITPLTPLISQ------
LdE93   ----SVITKIERAKSESDMETEDS------PS-NVILKIPSFRPTSSKNGCDIFRNPVEATGSMISPPVT----------

SgE93   GQE--SSHVLNSSSNNSNESCSPPASNLVGKG-----------IGVSLRDVIAKSISQKFQPTDLPMSQKLLLSAD----
BgE93   PES--SSQQL-------SDSCSPPVPSLVGKG-----------IGVSLRDVIAKSISQKFQQHS-ELSPKLGMVPE----
DmE93   NNSNHSSNSHRNGSNRSPHSASPMLAAAVAQGGYSAGNSLLTSSSSSIQKMMASNIQRQINEQSGQESLRNGNVSDCSSN
Bm2E93  PPS--NLLLSP------SVFADPPAGSQH--------------IFTSLNDVIAKSISQKFQQ---PLDRTHQ--------
BmE93   ----------------------PPAGSQH--------------IFTSLNDVIAKSISQKFQQ---PLDRTHQ--------
LdE93   -----------------SESGSPPIMPNKGL------------IIKDVKDVIAQSISQKFQQ---SLEPRRPI-------

SgE93   ----EQPPFKR----------GRFTPPLNTNITSCT-IKHNNNNNTNVDDKNK-------------TSSAGGKTSSNSTG
BgE93   ----TEPPFKR----------GRFTPPLVAGASATSVIKHNNNNNSQADDRNAQKILPQVQSKPTGTSSGASSQSSSSGG
DmE93   NGGSSSLGYKKPSISVAKIIGGTDTSRFGASPNLLS-QQHHSAHHLTHQQQQQ------------------QLSAQEALG
Bm2E93  ----ADLSFMR--------------APDARHVSVIK-SQSDNQRNYAMPSNSK--------------VPTNNNGQAAAGG
BmE93   ----ADLSFMR--------------APDARHVSVIK-SQSDNQRNYAMPSNSK--------------VPTNNNGQAAAGG
LdE93   ----IEVDFKR----------GRFTPPLGAGISVIK-TQHEMNRQYQPPPKPQ---------------QHLNDTTTTTGG

SgE93   KGTRPKRGKYRNYDRDSLVEAVRAVQRGEMSVHRAGSYYGVPHSTLEYKVKERHLMRPRKREPKQQQSDEVKRKDESGTS
BgE93   KGTRPKRGKYRNYDRDSLIEAVRAVQRGEMSVHRAGSHFGVPHSTLEYKVKERHLMRPRKREPKPPMEDAKKKEE-----
DmE93   KGTRPKRGKYRNYDRDSLVEAVKAVQRGEMSVHRAGSYYGVPHSTLEYKVKERHLMRPRKREPKPQPDLVGLT-------
Bm2E93  KGTRPKRGKYRNYDRDSLVEAVKAVQRGEMSVHRAGSYYGVPHSTLEYKVKERHLMRPRKREPKPPPQDTKPQ-------
BmE93   KGTRPKRGKYRNYDRDSLVEAVKAVQRGEMSVHRAGSYYGVPHSTLEYKVKERHLMRPRKREPKPPPQDTKPQ-------
LdE93   KGTRPKRGKYRNYDRDSLVEAVRAVQRGEMSVHRAGSYYGVPHSTLEYKVKERHLMRPRKRDPRPNPVDEKLATLK----

SgE93   IPRSTATTSSALANDKLKTLPKP-PK--------------TAFN-SPAPLPGAPNGLKIPPLFDPS-LSLAAYANVTTAF
BgE93   ------TRHPAATLEKSKIPPKSTPK--------------TPYTPSSSAIPSAPNGLKLPPMFDPANVPPMPYAT-APPF
DmE93   ------GPANKLQLDKLKAGPHGGSKLSNALKNQNNQAAAAAAAAAAAAAATPNGLKL-PLFEAGPQALS-----FQPN
Bm2E93  -------------------PPKPLPP--------------KP--------PGKP--------FSNGLNGPETPTY-PAGY
BmE93   -------------------PPKPLPP--------------KP--------PGKP--------FSN---------------
LdE93   ------QNDIRLAQDKMKPMMKP-PQ--------------QKYP------PTSPNGMKL-PIFEPGMTPLAGYN--APPF

SgE93   PFW-PGP-FHALPVPDFTTPTGSFPPNPEHFFAPQMIHRLQDETRTAPRTSPSSVVPPLGKTAREMAASLYDGTGANGNF
BgE93   PFWPPNP-FHSLPLP----PAG-FSPTQD-FFASQMMQRLGGGASSPAALSTSSRSPPLGKSAREMAESLYDGTGANGSF
DmE93   MFWPQTNATNAYGLD---------------------FNRITEAMRNPQASNHH----GLMKSAQDMVENVY---------
Bm2E93  PFW-ASPGFA--------------PPPTPDLYASHMMRRLREEAPPP----------------------------ANGSF
BmE93   --------------------------------------------------------------------------------
LdE93   PFWPHHPGFAHLPLDYARNPTSPFPPPSPDFFASQMMQKLQEESSRTMSAVPS---PALAKNARQMAESLLEGPGANGSF

SgE93   LDGIIRSSLEM----GLPSSSPKEGSTGVG-NSGKMSNKALIDQLCRNSCVTPLPKAPTPFSMKDCSTSVNSSDEEPVKQ
BgE93   LDGIIRSSLEM----GLPPGVPKEAE--------NMSNKALLDQLCRNSRLTPLPRAAVTL----IDGGASSSDEDSIKR
DmE93   -DGIIRKTLQASEGNGSAAGNGSNGSNGNGHGHGHGHGHALLDQLLVKKTPLPFTNHRN----NDYAATCSSASGESVKR
Bm2E93  LEGIIRSSLER-------------------------PGAALMQRLASE--------------------------------
BmE93   --------------------------------------------------------------------------------
LdE93   LDGIIRSSLES----GVPVSEEKSSNEEKNLAPENMSNKALLDQLCRNSRLTPLSKPAA------TTDGNSSGDESYAKG

SgE93   ES----VIPEQPLESASPAEPVVCLSNSSSGLPEHDCRIDSEHVTSVPDEKHSKDSVLPLSGVHCVSSTEDSSELFTKDS
BgE93   SSSRNAFTCSRATESDASTSAVVDLSPSSNGSTVERKR----HI----DEEGEEETMSP-------SATKPEDAEFSQET
DmE93   SG-------SPMGNYADIKRERLSADSGGSSDEEHSASHINNNNSDLAHNKNKSGGGGGGGGNGQTNGNGRSSRMTSRDD
Bm2E93  -----------------------------------------------------------------------SPSLVRRLA
BmE93   --------------------------------------------------------------------------------
LdE93   LS---------PLNFATGASHEDNNDDDSSSRYEKDV----------------------------------SDVNAIELS

SgE93   SEFHKEHVHNPT-----------EESACDSKTVKILGDIEAAKDLLP-------KEEG--------NEDSEQTEDTKSMT
BgE93   NGISNDNDNNATEDTDGDNDDNDEESACDSKNITKRVDEDAAKDDKQ-------DDAP--------EEEIEENEETKS-K
DmE93   SETDASSLKSGE-----------SGGQQNHKMMDLNGGSSSSSHIKCESEAATGHHSPGHHTTSILHEKLAQIKAEQVDQ
Bm2E93  ADVEEPAARRPR-----------LDSSDHQLAAEMREAVQRLRAD---------KLRP--------RNGTPAPSPPPAPP
BmE93   ------------------------------------------------------KLRP--------RNGTPAPSPPPAPP
LdE93   NDSNASSERKAT-----------DETERKQPRIYLNQDLAKPENLKPEMLVRFREALD--------HNGLNESSASENDG

SgE93   VNS-
BgE93   INS-
DmE93   ADQL
Bm2E93  DRA-
BmE93   DRA-
LdE93   PQD
```

Supplementary Figure S3: Multiple sequence alignment of *Sg*E93 with other E93 proteins. The multiple sequence alignment of *Sg*E93 (BBA65756.1) with E93 proteins from the German cockroach, *Blattella germanica* (*Bg*E93, CCM97102.1), the fruit fly, *Drosophila melanogaster* (*Dm*E93, NP_652002.2), the silkworm, *Bombyx mori* (*Bm*E93 and *Bm*2E93 sequences were obtained from

AIL29268.1 and the ORF of ICPK01035599.1, respectively) and the Colorado potato beetle, *Leptinotarsa decemlineata* (*Ld*E93, AQN67828.1). The sequences were aligned with MUSCLE and colored by 70% consensus using MView.Several domain structures for E93 are shown: the two HTH-DNA binding motifs, RHF1 (highlighted in yellow) and RHF2 (highlighted in blue), the CtBP-interaction motif (CtBP-im, highlighted in pink) and the NR-boxes (highlighted in grey).

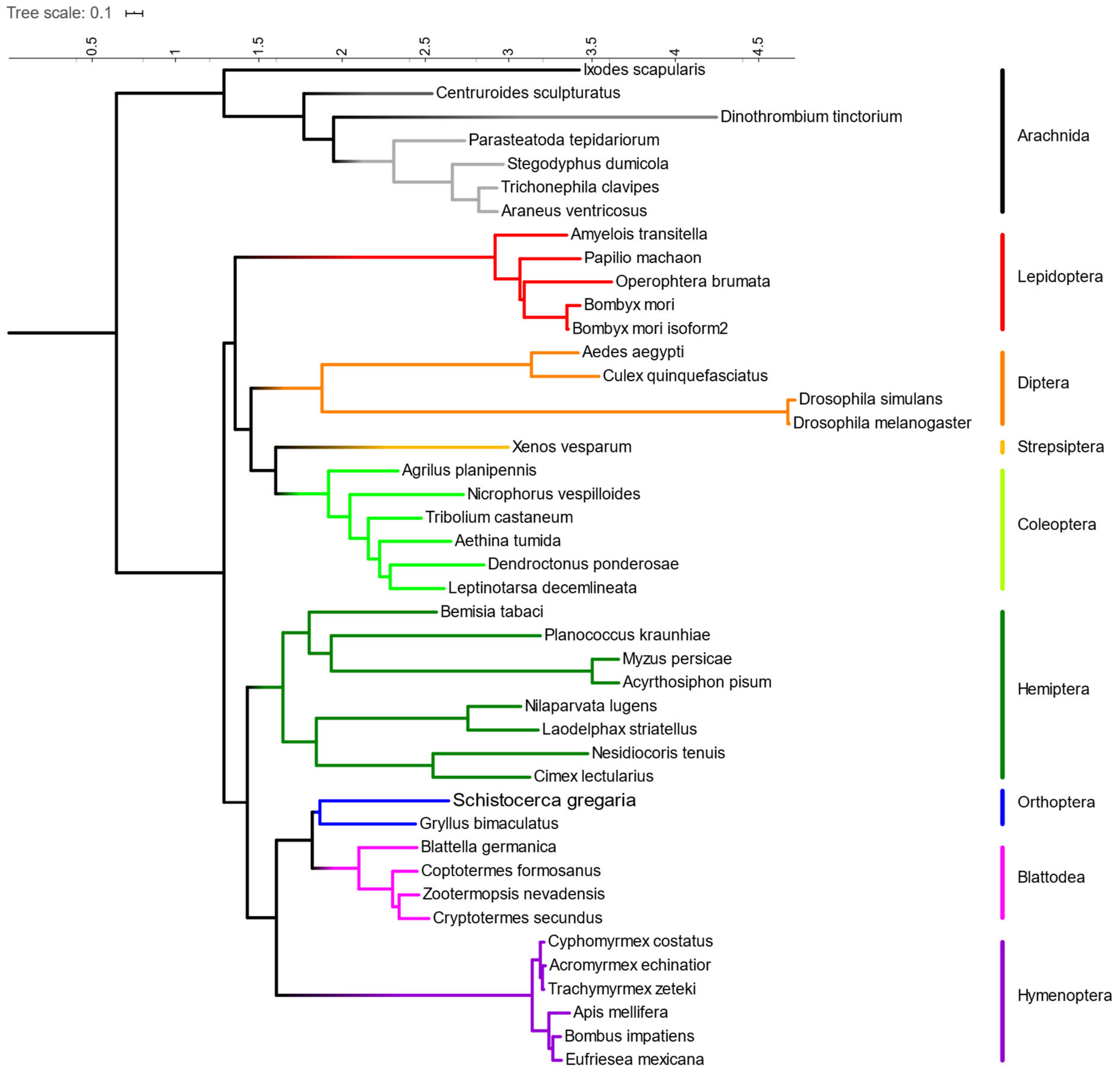

Supplementary Figure S4: phylogenetic analysis of E93 orthologs from insect and arachnid species. E93 sequences listed in Supplementary Table S1 were aligned with MUSCLE and used for a maximum likelihood analysis using IQ-tree. The resulting tree was rooted using the Arachnida class branch as the outgroup. The branches are colour coded according to the insect order. Scale values indicate evolutionary distance. Tree rendered with iTol.

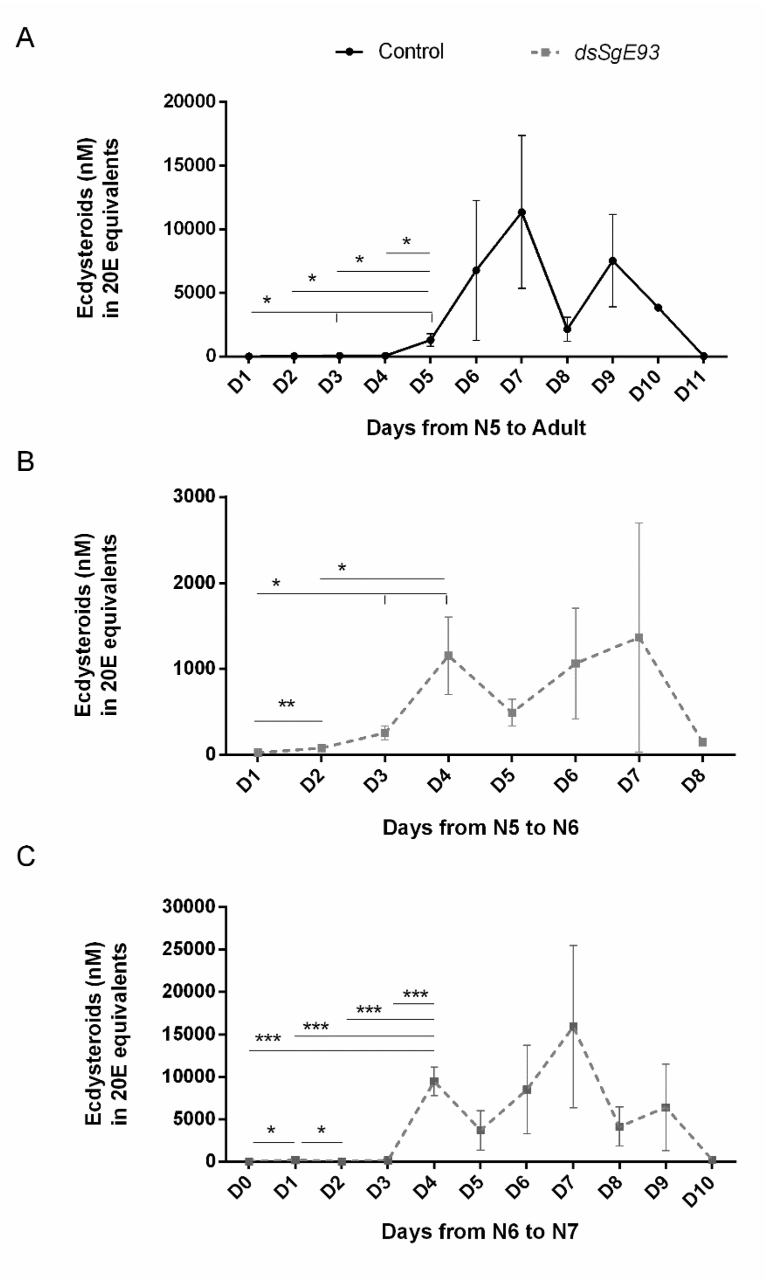

Supplementary Figure S5. Temporal ecdysteroid profiles in haemolymph of *dsSgE93*- and *dsGFP*-injected desert locust nymphs. Ecdysteroid levels (20E equivalents in nM) were measured in haemolymph samples that were collected daily from *dsGFP*-injected (control) fifth nymphal (N5) locusts (A), as well as *dsSgE93-injected* (*Sg*E93 knockdown) fifth nymphal (N5) (B) and sixth nymphal (N6) (C) locusts. Data are shown as the number of days after the previous (N4-to-N5) moult. Each data point in the figure represents mean ± S.E.M of 5 control (*dsGFP*) or 6 experimental (*dsSgE93*) locusts. Statistically significant differences between the measurements (starting on D0/1 until the following ecdysteroid peak) were found via a t-test (with or without two-sided Welch's correction) and are indicated by (an) asterisk(s) (* $p < 0.05$; ** $p < 0.01$; *** $p < 0.001$).

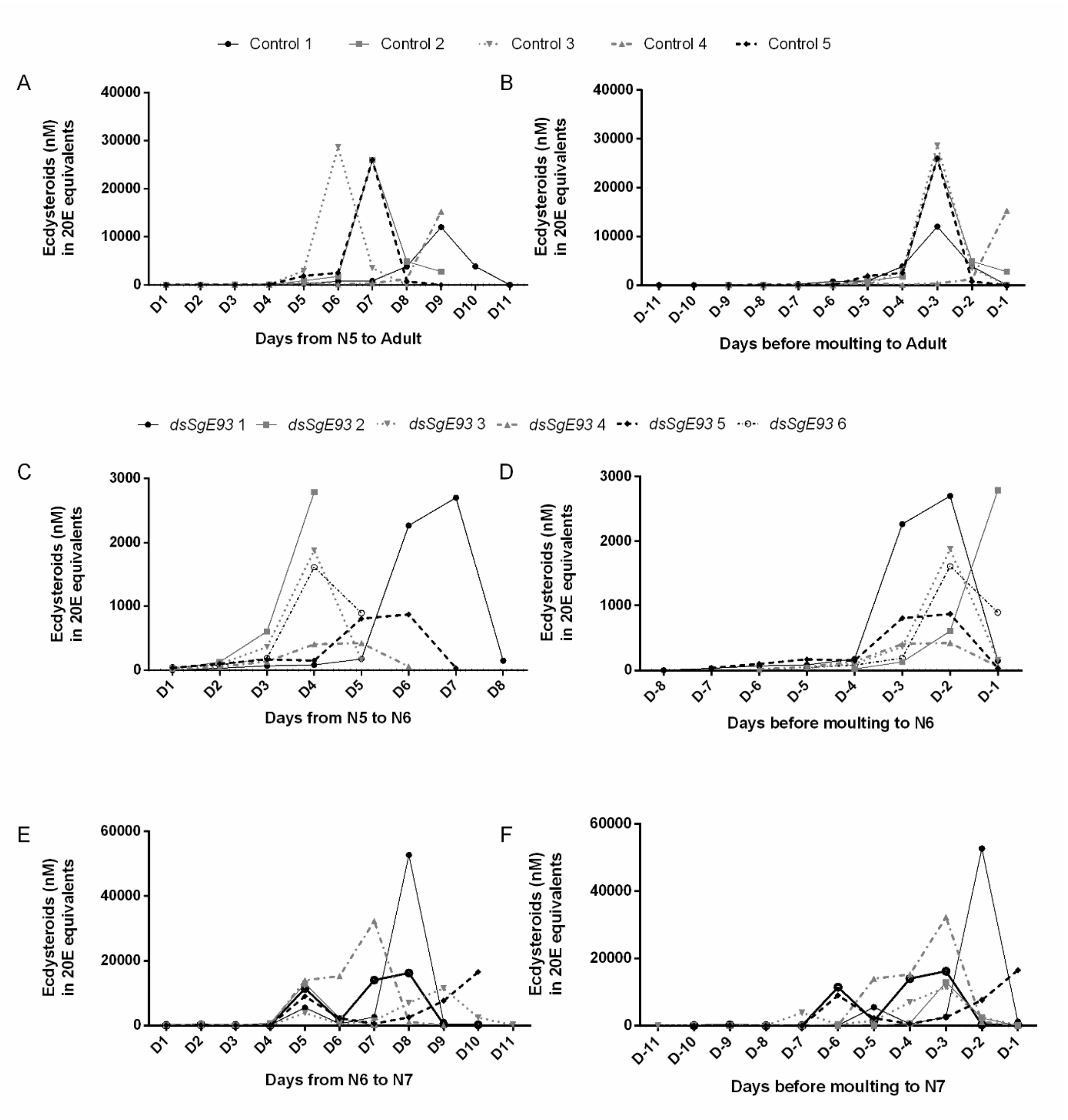

Supplementary Figure S6: Temporal ecdysteroid profiles in haemolymph of control (*dsGFP*) and *SgE93* knockdown (*dsSgE93*) nymphs. Ecdysteroid levels (20E equivalents in nM) were measured in the haemolymph of *dsGFP*- (control) and *dsSgE93*- (knockdown) injected locusts in the fifth nymphal stage (A - D) and of *dsSgE93*- (knockdown) injected locusts in the sixth nymphal stage (E + F). Each data point represents a measurement and each line represents an individual locust. In total, haemolymph samples from 5 control (*dsGFP*) and 6 experimental (*dsSgE93*) locusts were analysed.

Supplementary Table S1: Species and accession numbers of sequences used for constructing the phylogenetic tree shown in Suppl. Fig. S4.

**Species:**	**Accession number:**
Acromyrmex echinatior	EGI58724.1
Acyrthosiphon pisum	XP 008189864.1
Aedes aegypti	EAT44017.1
Aethina tumida	XP 019872192.1
Agrilus planipennis	XP 018321151.1
Amyelois transitella	XP 013188059.1
Apis mellifera	BAB64310.1
Araneus ventricosus	GBL83064.1
Bemisia tabaci	XP 018900968.1
Blattella germanica	CCM97102.1
Bombus impatiens	XP 003490553.1
Bombyx mori	AIL29268.1
Bombyx mori isoform 2	ICPK01035599.1 derived
Centruroides sculpturatus	XP 023227973.1
Cimex lectularius	XP 014260111.1
Coptotermes formosanus	GFG28607.1
Cryptotermes secundus	XP 023704791.1
Culex quinquefasciatus	XP 001863525.1
Cyphomyrmex costatus	KYN00623.1
Dendroctonus ponderosae	XP 019771885.1
Dinothrombium tinctorium	RWS15376.1
Drosophila melanogaster	NP 652002.2
Drosophila simulans	EDX13858.1
Eufriesea mexicana	XP 017758118.1
Gryllus bimaculatus (partial)	BBJ70113.1
Ixodes scapularis	EEC08637.1
Laodelphax striatellus	RZF34802.1
Leptinotarsa decemlineata	AQN67828.1
Myzus persicae	XP 022167663.1
Nesidiocoris tenuis	CAA9993019.1
Nicrophorus vespilloides	XP 017770390.1
Nilaparvata lugens	APO13600.1
Operophtera brumata	KOB75508.1
Papilio machaon	KPJ20458.1
Parasteatoda tepidariorum	XP_015926289.1
Planococcus kraunhiae	BBD13408.1
Schistocerca gregaria	BBA65756.1
Stegodyphus dumicola	XP 035205796.1
Trachymyrmex zeteki	KYQ50221.1
Tribolium castaneum	KYB25179.1
Trichonephila clavipes	PRD31503.1
Xenos vesparum	AYD60146.1
Zootermopsis nevadensis	KDR22086.1

Supplementary Table S2: Oligonucleotide sequences of primers used for qRT-PCR.

Reference/ target genes	Forward primer	Reverse primer
SgAct	5’- AATTACCATTGGTAACGAGCGATT -3’	5’- TGCTTCCATACCCAGGAATGA -3’
SgEF1α	5’- GATGCTCCAGGCCACAGAGA -3’	5’- TGCACAGTCGGCCTGTGAT -3’
SgE93	5’- CGCAAGCAGACATAGAACCC -3’	5’- TGGTCTCAGGAACATCCCAC -3’
SgMet	5’- GGTGCCTGAAGAGGAAGAAA -3’	5’- ATGGAGGTGATGAAGGAGAAAG -3’
SgKr-h1	5’- CTCCAAGACGTTCATCCAGAG -3’	5’- TGCTTGGAGCAGGTGAAG -3’
SgCYP15A1	5’- AAAGCAACTTCATCATTCACAGATG -3’	5’- CAGAGCCAGCCATGAACAAA -3’
SgJHAMT	5’- CGGAGCAAAGGCAAGCA -3’	5’- CCACTTCACCGCCTGGTTT -3’
SgSpo	5’- CAACATCTTCACCAGCTACATGTG -3’	5’- GGGTCGTCGTAGTCGAAGGA -3’
SgPhm	5’- CGCAGAGCCCGGACAAC -3’	5’- CGAACATGTCGGCCATGA -3’
SgSad	5’- ATCGTGGCCGAGATTACGAA -3’	5’- AGCACCATCTCCGGATCCT -3’
SgDib	5’- CCCAGGCTGCTATCGAGACT -3’	5’- CGACGACCAGGCCTATGTAGTT -3
SgEcR	5’- AAGGTTGATAATGCGGAATATGC -3’	5’- GTGATGGGCGCTCTGAAAAT -3’
SgRXR	5’ -AATGCCTCGCTATGGGAATG -3’	5’- TCCTTTGTTCGCTGCCTTTC -3’

Abbreviations: *Sg* = *Schistocerca gregaria*; *Act* = *β-actin*; *EF1α* = *Elongation factor 1α*; *Met* = *Methoprene-tolerant*; *Kr-h1* = *Krüppel-homolog 1*; *CYP15A1* = *Cytochrome P450 enzyme 15A1 (methyl farnesoate epoxidase)*; *JHAMT* = *Juvenile hormone acid methyltransferase*; *Spo* = *Spook*; *Phm* = *Phantom*; *Sad* = *Shadow*; *Dib* = *Disembodied*; *EcR* = *Ecdysone receptor; RXR* = *Retinoid X receptor.*

Supplementary Table S3: Oligonucleotide sequences of primers used for dsRNA synthesis. Nucleotides indicated in ‘bold’ represent the T7 promoter sequences used for preparing the dsRNA constructs.

RNAi constructs	F-primer	R-primer
dsSgE93	5’- **TAATACGACTCACTATAGGGAGA** GCCCAAGCAACAACAGTCTG -3’	5’- **TAATACGACTCACTATAGGGAGA** GGCAAGCCCATCTCAAGACT -3’
dsGFP	5’-**TAATACGACTCACTATAGGGAGA** AAGGTGATGCTACATACGGAA -3’	5’-**TAATACGACTCACTATAGGGAGA** ATCCCAGCAGCAGTTACAAAC-3’

Abbreviations: *Sg* = *Schistocerca gregaria*; *GFP* = *Green fluorescent protein.*
